# Altering substrate availability profoundly affects left ventricular function in the normal heart

**DOI:** 10.1186/1532-429X-18-S1-Q32

**Published:** 2016-01-27

**Authors:** Andrew Lewis, Stefan Neubauer, Oliver Rider

**Affiliations:** grid.4991.50000000419368948University of Oxford, Oxford, United Kingdom

## Background

Although altered cardiac substrate selection has been proposed to be the cause for many cardiac diseases and has been suggested to be a target to improve cardiac function, the effect of modulating substrate availability on global LV function in the normal heart has not been investigated. Our aim was to use CMR to investigate global cardiac function and strain before and after modulation of substrate availability

## Methods

Six subjects (3 male) with no cardiac risk factors (age 23 ± 1 yrs, BMI 23.7 ± 1.8 kg/m^2^), underwent CMR imaging (1.5T) before and 4 hours after substrate manipulation. All measurements were performed after an overnight fast. Circulating free fatty acid (FFA) levels were elevated with a triglyceride infusion (60 mL/hr, 20% Intralipid™, Fresenius Kabi, U.K) co-administered with unfractionated heparin (0.4 U/kg per min). Reduction of circulating free fatty acids, and increased myocardial glucose uptake, was promoted with a single dose of nicotinic acid (Niaspam 400 mg). Analysis was undertaken in cvi^42^® with 1) tissue tracking to derive global LV measures of systolic radial and circumferential strain (%), diastolic radial strain rate (%/s), and 2) left ventricular ejection fraction (LVEF, %).

## Results

All subjects had normal LV function at rest (LVEF range 57-79%). Intralipid infusion resulted in elevation of; circulating FFA (baseline 0.16 ± 0.07 vs 4 hr 1.38 ± 0.18 mM, p = 0.01), B-hydroxybutyrate levels (0.26 ± 0.06 vs 0.41 ± 0.08 mmol/l, p = 0.004), systolic blood pressure (SBP 109 ± 11 vs 116 ± 8 mmHg, p = 0.04), and a fall in glucose levels (4.6 ± 0.7 vs 3.7 ± 0.7 mmol/l, p = 0.021). In every subject, this was associated with an increase in LVEF, (64.8 ± 6.6 vs 69 ± 7.2%, p = 0.006), and improved systolic peak global radial (34.2 ± 7.8 vs 38.3 ± 8.3%, p = 0.003) and circumferential (-18.9 ± 2.6 vs -20.2 ± 2.6%, p = 0.001) strain. In contrast, nicotinic acid caused a fall in; circulating FFA (0.24 ± 0.15 vs 0.10 ± 0.23 mM, p= 0.045) with no change in B-hydroxybutyrate (0.39 ± 0.36 vs 0.27 ± 0.15 mmol/l, p = 0.54), glucose (3.9 ± 0.9 vs 3.7 ± 0.7 mmol/l, p = 0.34) or SBP (112 ± 13 vs 107 ± 8 mmHg, p = 0.21). In almost every subject, this was associate with decreased LVEF (66.6 ± 7.1 vs 61.8 ± 7.2%, p = 0.007), and impairment of both systolic peak global radial (44.4 ± 18.5 vs 35.5 ± 16.0%, p = 0.015) and circumferential strain (-21.2 ± 4.5 vs -18.7 ± 4.9%, p = 0.01). Diastolic strain rate also worsened with nicotinic acid (-369 ± 147 vs -227 ± 119%/s, p = 0.018).

## Conclusions

In the fasting state**,** LV function in the normal heart is affected substantially by altering substrate availability. Whilst elevating FFA acutely results in increased LVEF and improved systolic strain, in the same hearts, lowering FFA and potentially increasing glucose uptake with nicotinic acid results in reduced LVEF and impaired systolic and diastolic strain. This suggest that even in the normal heart cardiac altering substrate selection can substantially affect LV function.

**Figure 1:**
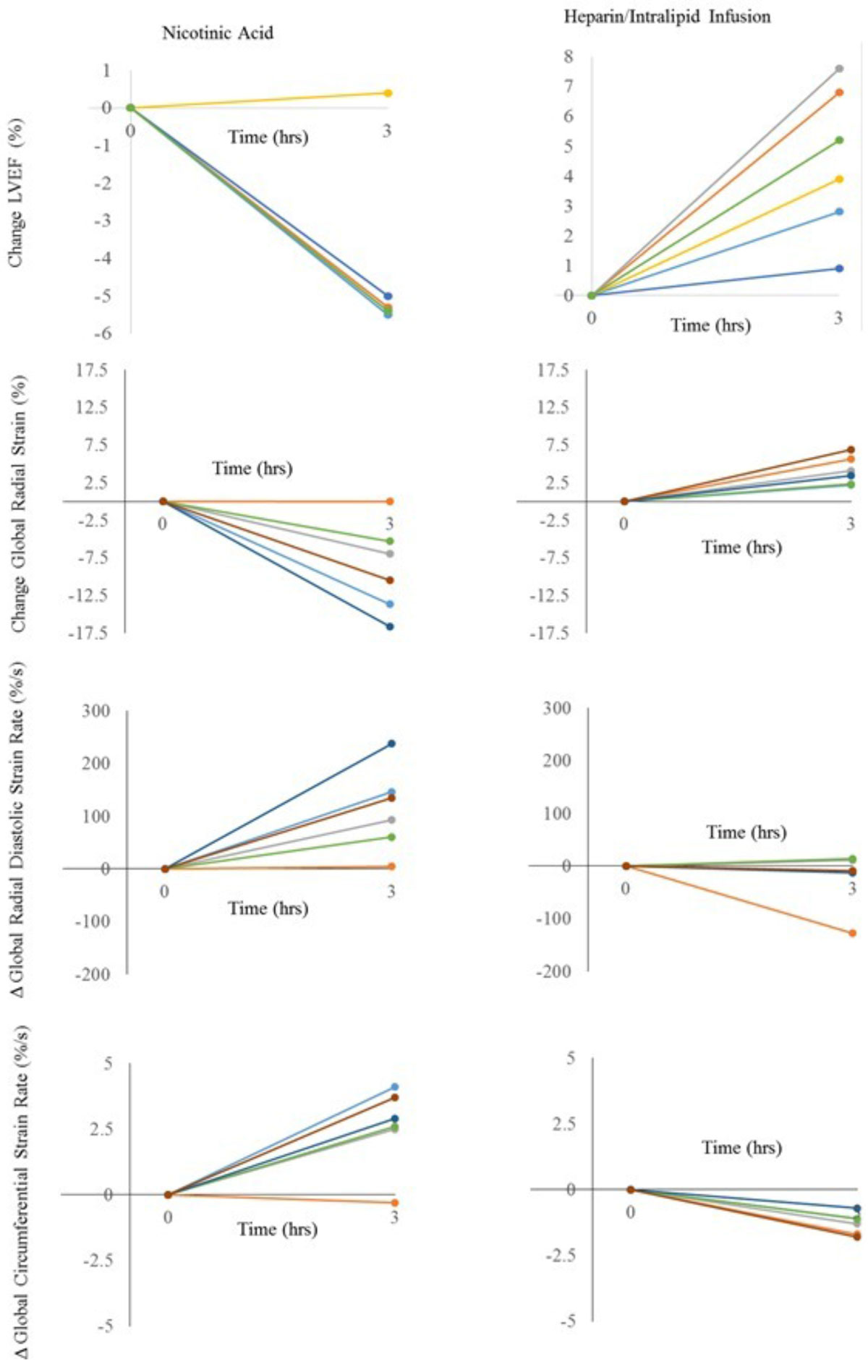
Figure 1

